# Antibody engineering to generate SKY59, a long-acting anti-C5 recycling antibody

**DOI:** 10.1371/journal.pone.0209509

**Published:** 2018-12-28

**Authors:** Zenjiro Sampei, Kenta Haraya, Tatsuhiko Tachibana, Taku Fukuzawa, Meiri Shida-Kawazoe, Siok Wan Gan, Yuichiro Shimizu, Yoshinao Ruike, Shu Feng, Taichi Kuramochi, Masaru Muraoka, Takehisa Kitazawa, Yoshiki Kawabe, Tomoyuki Igawa, Kunihiro Hattori, Junichi Nezu

**Affiliations:** 1 Research Division, Chugai Pharmaceutical Co., Ltd., Gotemba, Shizuoka, Japan; 2 Research Division, Chugai Pharmaceutical Co., Ltd., Kamakura, Kanagawa, Japan; 3 Chugai Pharmabody Research Pte. Ltd., Singapore, Singapore; ChemPartner, CHINA

## Abstract

Modulating the complement system is a promising strategy in drug discovery for disorders with uncontrolled complement activation. Although some of these disorders can be effectively treated with an antibody that inhibits complement C5, the high plasma concentration of C5 requires a huge dosage and frequent intravenous administration. Moreover, a conventional anti-C5 antibody can cause C5 to accumulate in plasma by reducing C5 clearance when C5 forms an immune complex (IC) with the antibody, which can be salvaged from endosomal vesicles by neonatal Fc receptor (FcRn)-mediated recycling. In order to neutralize the increased C5, an even higher dosage of the antibody would be required. This antigen accumulation can be suppressed by giving the antibody a pH-dependent C5-binding property so that C5 is released from the antibody in the acidic endosome and then trafficked to the lysosome for degradation, while the C5-free antibody returns back to plasma. We recently demonstrated that a pH-dependent C5-binding antibody, SKY59, exhibited long-lasting neutralization of C5 in cynomolgus monkeys, showing potential for subcutaneous delivery or less frequent administration. Here we report the details of the antibody engineering involved in generating SKY59, from humanizing a rabbit antibody to improving the C5-binding property. Moreover, because the pH-dependent C5-binding antibodies that we first generated still accumulated C5, we hypothesized that the surface charges of the ICs partially contributed to a slow uptake rate of the C5–antibody ICs. This idea motivated us to engineer the surface charges of the antibody. Our surface-charge engineered antibody consequently exhibited a high capacity to sweep C5 and suppressed the C5 accumulation *in vivo* by accelerating the cycle of sweeping: uptake of ICs into cells, release of C5 from the antibody in endosomes, and salvage of the antigen-free antibody. Thus, our engineered anti-C5 antibody, SKY59, is expected to provide significant benefits for patients with complement-mediated disorders.

## Introduction

Antibody engineering is in high demand for generating therapeutic antibodies with higher efficacy, safety, and convenience for patients than can be achieved by conventional antibodies. Antibody engineering and optimization technologies can improve various properties of therapeutic antibodies, such as their antigen-binding properties, pharmacokinetics (PK), pharmaceutical properties, immunogenicity, and effector functions [1–4]. However, while high affinity antibodies have been shown to be therapeutically effective, some require a huge, often unrealistic, dosage [5–7]. In theory, even if an antibody has infinite affinity, it would still need to be at a higher concentration than the total antigen to be able to completely neutralize the antigen *in vivo*.

Antigen accumulation in plasma is a phenomenon observed when an antibody that targets a soluble antigen in circulation is administered, and it requires a higher dose of the antibody to be administered to achieve sufficient efficacy [7]. This accumulation occurs because the antigen is synthesized continuously *in vivo* but the clearance of the antigen is reduced by the administered antibody [8,9], which generally has a long plasma half-life because of neonatal Fc receptor (FcRn)-mediated recycling [10]. The property by which an antibody binds to an antigen pH dependently has been studied strenuously in recent years [11–25] in hopes of achieving longer-lasting efficacy and a lower dose for treatment by suppressing the antigen accumulation. An antibody that binds to its antigen pH-dependently, also known as a *recycling* or *sweeping* antibody, is capable of dissociating its bound antigen in the acidic endosome, which lets the antigen-free antibody be recycled back to plasma by the FcRn-mediated recycling. This allows the antibody to bind to its target antigen multiple times, which in turn reduces the amount of the antibody required for treatment. However, even a pH-dependent antibody causes soluble antigens to accumulate, if the uptake of the immune complex (IC) is too slow. This source of antigen accumulation can be overcome by increasing the interaction of the antibody with Fc receptors so that the uptake of ICs by cells is accelerated [12,14,24]. However, the enhanced interaction with Fc receptors can also increase the uptake of antigen-free antibodies, resulting in larger clearance of the antibody. Hence, engineering that can further suppress the antigen accumulation without compromising the long plasma half-life of the antibody is still desired.

The complement system is an important immune system, and because uncontrolled complement activation is known to lead to a severe pathology, modulating the complement system is currently recognized as a promising strategy in drug discovery [26]. The complement cascade can be activated through any of the three pathways, namely the classical, lectin, and alternative pathways. They all converge in one final common pathway in which complement C5 is cleaved into C5a and C5b. The anaphylatoxin C5a is a potent inflammatory mediator, and C5b initiates the assembly of the membrane attack complex (MAC) for cell lysis. Hence, inhibiting the cleavage of C5 is thought to be the most effective approach in regulating abnormal complement activation. Eculizumab, a humanized monoclonal antibody that blocks the cleavage of C5, has demonstrated clinical efficacy in the treatment of complement-mediated diseases such as paroxysmal nocturnal hemoglobinuria (PNH) [5,27], atypical hemolytic uremic syndrome (aHUS) [28], and generalized myasthenia gravis [29]. However, because the concentration of C5 in plasma is high (approximately 80 μg/mL) [30], patients need a large amount (900–1200 mg) of eculizumab every other week, and this huge dosage requires intravenous administration. Moreover, it has been demonstrated that the polymorphism R885H in C5 leads to a poor response to eculizumab treatment [31]. Recently, we generated SKY59, a humanized anti-C5 monoclonal antibody with the pH-dependent antigen-binding property that is crucial for reducing the required dosage, and found that its activity was not affected by the rare R885H genetic polymorphism in C5 [17]. Although long-lasting neutralization of C5 by SKY59 has been reported, the details of the antibody engineering incorporated in this molecule have yet to be described.

Here we report the antibody engineering performed to generate SKY59. An anti-C5 antibody, CFA0305, with a pH-dependent C5-binding property, was directly identified through rabbit immunization [17]. After humanization, we identified the mutations that improved the C5-binding property by taking the approach of comprehensive substitution for multidimensional optimization (COSMO). We also found non-histidine mutations that further improved the pH dependency of the C5–antibody interaction. Although we achieved an antibody variant with improved pH-dependent binding to C5 and moderately enhanced FcRn binding at acidic pH for a longer plasma half-life [32], this variant significantly increased the plasma C5 concentration. We hypothesized that the IC uptake rate was too slow, which motivated us to use a novel type of antibody engineering to suppress the C5 accumulation without strongly affecting the antibody PK. This novel type of engineering applies mutations that modify the surface charge or isoelectric point (pI) of the antibody to achieve the optimal balance between antibody PK and C5 clearance that was reported for SKY59 [17]. Without compromising the long plasma half-life of SKY59, this engineering successfully suppressed C5 accumulation by accelerating the cycle of C5 sweeping; a cycle through which ICs are taken up into cells, C5 is dissociated from SKY59 in the endosome, and C5-free SKY59 is salvaged back to plasma. This modification can further reduce the required dosage of a pH-dependent antigen-binding antibody for treatment and can be used on a wider basis when antigen accumulation needs to be suppressed. We also engineered the antibody to have less immunogenicity and better pharmaceutical properties.

## Materials and methods

### Preparation of recombinant C5 and antibodies

Recombinant human C5 and cynomolgus monkey C5 were used for experiments unless stated otherwise. Human or cynomolgus C5 was transiently expressed using FreeStyle 293-F cells (Thermo Fisher Scientific) and purified as reported previously [17]. Recombinant antibodies were also transiently expressed using FreeStyle 293-F cells and purified with a conventional method using MabSelect SuRe (GE Healthcare) or Ab-Capcher (ProteNova). Gel filtration was further conducted if necessary.

### Comprehensive mutagenesis

To identify mutations that are effective or useful for engineering our lead antibodies, we took the COSMO approach, in which all residues in the complementarity-determining regions (CDRs) and some key residues in framework regions (FRs) were substituted with a different natural amino acid except cysteine. Eighteen antibody variants were expressed to evaluate the 18 single mutations (each of the natural amino acids except the original one and cysteine) at each of the residues that could directly or indirectly contribute to the C5 binding; therefore, over 1,000 antibody variants were expressed. To produce a huge number of antibodies, we made a series of polymerase chain reaction (PCR) products, each containing an antibody-expression cassette with a single mutation. FreeStyle 293-F cells (Thermo Fisher Scientific) were then transfected using the PCR products for a heavy or light chain variant with a mutation and plasmids for the other chain with no mutation. Antibody variants expressed by the transfected cells were purified by protein A in a 96-well plate format, if necessary. In this way, it is now possible to produce and evaluate over 1,000 antibody variants within a few weeks, whereas producing this huge number of antibody variants by the conventional method using plasmid vectors takes several months.

### Binding kinetics analysis by surface plasmon resonance (SPR)

The binding kinetics of anti-C5 antibodies to C5 was assessed at pH 7.4 and pH 5.8 at 37°C using a Biacore T200 instrument (GE Healthcare). Each antibody was captured onto the anti-human Fc (GE Healthcare) that was immobilized on a CM4 sensor chip (GE Healthcare), after which recombinant C5 was injected over the flow cells. The sensor chip surface was regenerated using 3 M MgCl_2_. Kinetic parameters were determined by fitting the sensorgrams with 1:1 binding model using Biacore T200 evaluation software, version 2.0 (GE Healthcare). The pH-dependent C5-binding property of each antibody was evaluated by a modified Biacore assay, in which an additional dissociation phase at pH 5.8 was integrated immediately after the dissociation phase at pH 7.4. The dissociation rate at pH 5.8 was determined by processing and fitting data using Scrubber 2.0 curve fitting software (BioLogic Software) to assess the pH-dependent dissociation of C5 and antibodies from the complexes formed at pH 7.4.

### *In vivo* study using human FcRn transgenic mice

Animal care and experiments were performed in accordance with the guidelines for the care and use of laboratory animals at Chugai Pharmabody Research (CPR). The experimental protocols were approved by Institutional Animal Care and Use Committee of CPR and all the experimental procedures were carried out by licensed personnel. Twelve human FcRn homozygous transgenic mice, line #32 (B6.16 mouse FcRn–/–, human FcRn transgenic line 32 +/+ mouse, Jackson Laboratories), were used in this study. The mice were housed (3 mice/cage) with food, water, and enrichments (wood blocks and a house for nesting). All mice were observed twice a day. The mice were euthanized at the end of the study via CO_2_ inhalation. Human C5 prepared from serum (Merck Millipore) was intravenously administered with or without anti-C5 antibodies to the mice, as single doses of 0.1 mg/kg for C5 and 20 mg/kg for the antibodies. The concentration of total human C5 or the anti-C5 antibodies in mouse plasma was measured as previously described [17].

### *In vivo* study using cynomolgus monkeys

Animal care and experiments were performed in accordance with the guidelines for the care and use of laboratory animals at Maccine, Pte. Ltd. (Singapore). The experimental protocols were approved by Institutional Animal Care and Use Committee of Maccine, and all the experimental procedures were carried out by licensed personnel. Twenty-four cynomolgus monkeys previously received from Vietnam were used in the study. The rooms were illuminated by fluorescent lights set on a 12-hour light-dark cycle and were air conditioned, with the temperature and relative humidity measured daily. The actual ranges for temperature and humidity were 19°C–25°C and 51%–70%, respectively. Animals were fed a diet of monkey chow free of animal protein that was offered twice daily, and a controlled amount of fruit was also offered twice daily. Mains tap water was offered *ad libitum*. The animals were provided with cage toys for environmental enrichment to ensure adequate welfare and psychological well-being. All animals were observed once daily. The animals were returned to stock on completion of the study. Anti-C5 antibodies were intravenously administered to cynomolgus monkeys. 305LO1-SG407, 305LO1-SG419, 305LO5-SG115, or 305LO8-SG115 (20 mg/kg) was administered at day 0. CFA0322-IgG_4_ (40 mg/kg) was administered at day 0 and 14. 305LO1-SG406 (40 mg/kg) was administered at day 0, 3, 6, and 9. The concentration of endogenous C5 or anti-C5 antibodies in cynomolgus monkey plasma was measured as previously described [17].

### Isoelectric point measurement

Isoelectric focusing was accomplished in PhastSystem (GE Healthcare) at 15°C. The pH gradient was formed on the PhastGel IEF media 3–10.5 (pH range 3–10.5) containing pharmalyte carrier ampholytes for 75 Vh at the voltage of 2000 V. Samples were applied to the gel for 15 Vh at 200 V, and they were then allowed to migrate to their isoelectric points for 410 Vh at 2000 V. After migration, the gel was fixed with fixing solution (20% TCA, Sigma Aldrich) followed by washing with 30% methanol and 10% acetic acid in distilled water. The staining was done in InstantBlue solution (Expedeon) for 1 hour followed by washing with distilled water until the desired background was achieved.

### Cation exchange chromatography analysis

Cation exchange (CIEX) chromatography analysis was carried out using ProPac WCX-10 column, 10 μm, 4 mm × 250 mm (Thermo Fisher Scientific) with mobile phase A (25 mM MES, pH 7.0) and mobile phase B (25 mM MES, 250 mM NaCl, pH 7.0) at a flow rate of 0.5 mL/min on Alliance HPLC System (Waters). A linear gradient condition was applied from 10% to 70% of B in 50 min. The column was then washed for 5 min at 100% of B and further equilibrated for 5 min at 10% of B. Elution was monitored by UV absorbance at 280 nm. For sample preparation of ICs, 0.2 μM of each antibody was mixed with 0.2 μM of cynomolgus C5 and incubated for 1 hour at 25°C in mobile phase A.

### *In silico* evaluation of immunogenicity

The potential immunogenicity of each antibody was evaluated *in silico* using Tregitope-adjusted EpiMatrix score (EpiVax) [33–35] and Epibase immunogenicity risk score (Lonza) [36].

## Results

### The research flow of identification and optimization of an anti-C5 antibody

**Fig 1:**
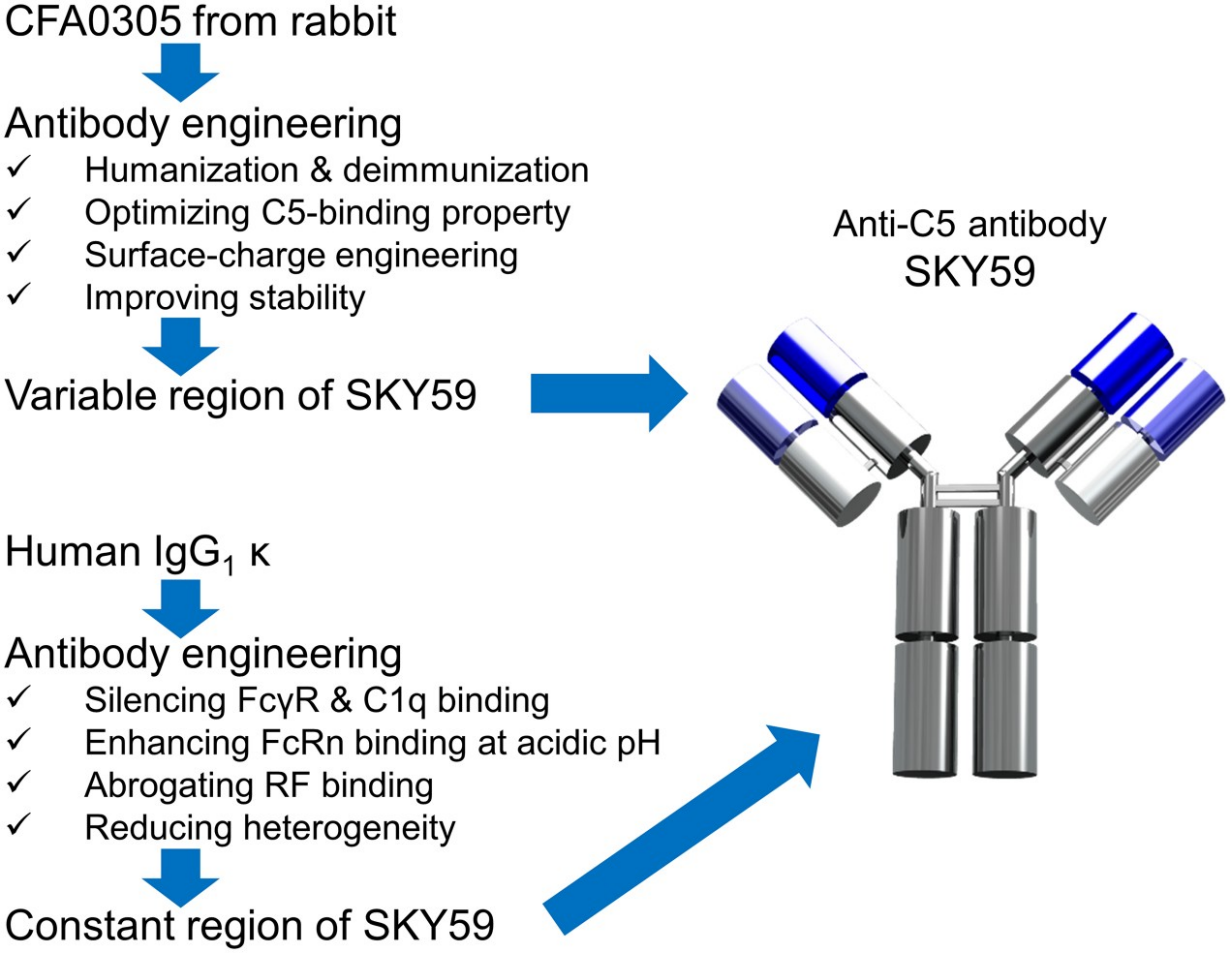
Flow to generate the long-acting anti-C5 antibody. The variable region of SKY59 was generated through rabbit immunization and multidimensional optimization (blue region). The constant region of SKY59 was designed by engineering a human IgG_1_ κ constant region (silver region).

The antibody generation and engineering flow is summarized in Fig 1. Anti-C5 antibodies were obtained from rabbits immunized with human C5 [17]. We identified a lead antibody, termed CFA0305, which inhibited the enzymatic cleavage of both human and cynomolgus C5. This antibody exhibited a favorable pH-dependent antigen-binding property of stronger C5 binding at pH 7.4 than at pH 5.8. To improve various properties of the antibody, the variable region of CFA0305 was humanized and multidimensionally optimized, and a human IgG_1_ κ constant region was also engineered. The engineered variable and constant regions were combined to generate SKY59 with ideal properties for clinical promise. The details are described in the following sections of this report.

### Humanization of the rabbit antibody and removal of cysteine residues in CDRs

The rabbit variable region (V_H_ and V_L_) of the lead antibody, CFA0305, was humanized to minimize the potential immunogenicity. In this humanization process, known as framework shuffling, each rabbit framework (FR1, FR2, FR3, and FR4) was individually replaced with a human germline framework that was considered to be structurally appropriate for its binding activity [37], resulting in a human V_H_3-V_H_2 hybrid heavy chain and a human V_κ_1 light chain. We found cysteine residues in the CDR-H1, CDR-H2, and CDR-L3. Because they may cause disulfide bond-related aggregation or heterogeneity, these cysteine residues were substituted with alanine or serine to promote the developability of the antibody, despite the fact that this engineering reduced the C5-binding affinity (data not shown). This antibody with the substitutions was chosen as the lead humanized antibody for further engineering.

### Comprehensive mutagenesis in the variable region to improve the C5-binding property

Although the lead antibody displayed pH dependency without engineering, its low affinity to C5 (*K*_D_ = 4.56 × 10^−9^ M) with a slow association rate constant (*k*_a_ = 9.52 × 10^4^ M^-1^ s^-1^) at pH 7.4 was considered to be insufficient to completely neutralize complement activity *in vivo* [17], and the lead humanized antibody with no extra cysteine in its CDRs showed even weaker affinity. In order to identify mutations that improve the C5-binding property, we expressed more than 1,000 antibody variants, each containing a single mutation through the COSMO approach, as described in Materials & Methods. In this way, we identified mutations that increased the association rate, which are crucial for our desired pH-dependent C5-binding antibody [16] but are difficult to identify by simple panning methods from display libraries. In parallel, we also identified mutations that did not affect the C5-binding property and could therefore be useful for further antibody engineering, such as surface-charge engineering, improving stability, and reducing immunogenicity.

**Fig 2:**
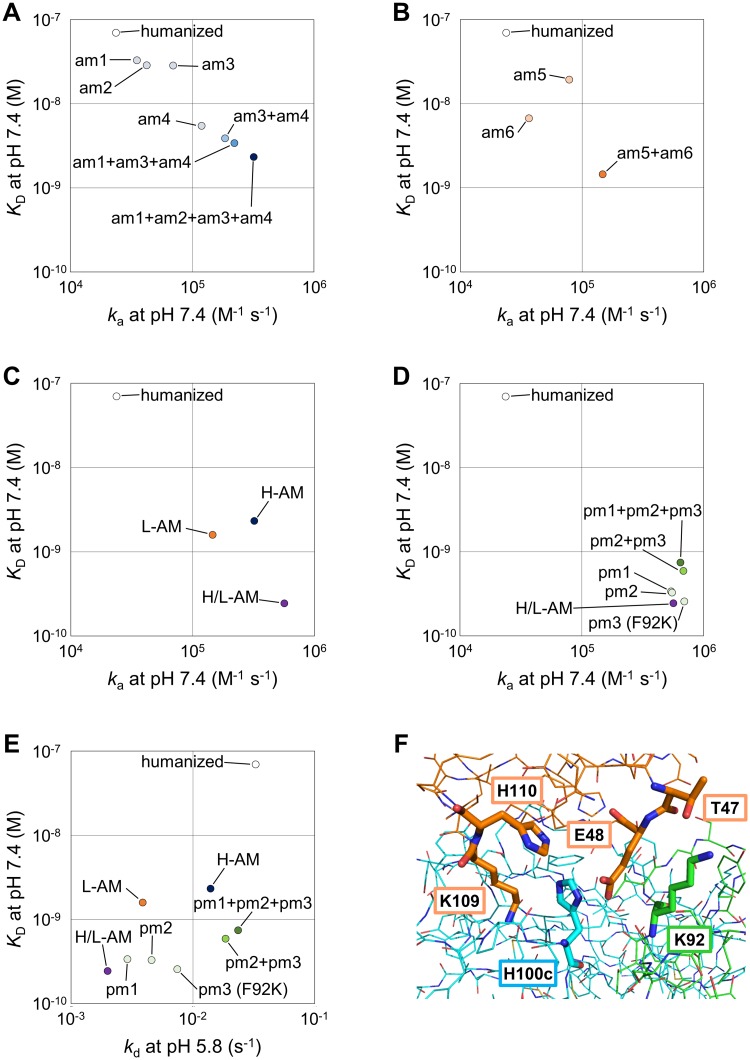
Effect of mutations and their combinations on the C5-binding property. C5-binding activity of the humanized antibody or each antibody variant containing single or multiple mutations was measured by Biacore. Mutations in the heavy chain (am1–am4) and the light chain (am5, am6) cumulatively improved the C5-binding affinity (A and B). The combination of an affinity-matured heavy chain (H-AM) with an affinity-matured light chain (L-AM) further enhanced the affinity (C). Non-histidine mutations found in the heavy chain (pm1, pm2) or the light chain (pm3), and their combinations did not worsen *k*_a_ at pH 7.4 (D) and provided even better pH dependency (E). The crystal structure of the interaction site of SKY59 and the MG1 domain in human C5 is shown using PyMOL (Schrödinger, LLC) (F). Orange, light blue, and light green represent human C5, the heavy chain, and the light chain of SKY59, respectively. The lysine introduced in the CDR-L3 to improve pH dependency, H100c in the CDR-H3, T47, E48, K109, and H110 in human C5 are shown with thick sticks. The structure of SKY59 and the MG1 domain complex has been shown previously [17] and registered in the RCSB Protein Data Bank with PDB ID: 5B71.

Derivatives of the lead humanized antibody that each contained single or multiple effective mutations were purified, and their C5-binding kinetics were evaluated by Biacore. Effects of the mutations listed in Table 1 or their combinations on *K*_D_ and *k*_a_ at pH 7.4 or *k*_d_ at pH 5.8 are depicted in Fig 2A–2E. Mutations for affinity maturation found in the heavy chain (am1–am4) and ones found in the light chain (am5, am6) cumulatively improved *K*_D_ and *k*_a_ at pH 7.4 (Fig 2A and 2B). The combination of the affinity-improved heavy and light chains exhibited even better *K*_D_ and *k*_a_ at pH 7.4 (Fig 2C). We also carefully monitored the effect of each mutation on the pH-dependent C5 binding and chose mutations that did not strongly affect the pH dependency. In addition, we found non-histidine mutations that improved the pH dependency in the heavy chain (pm1, pm2) and the light chain (pm3) by the COSMO approach. We then introduced these mutations in the affinity-matured antibody (H/L-AM), which resulted in increased *k*_d_ at pH 5.8 without strongly affecting *k*_a_ or *K*_D_ at pH 7.4 (Fig 2D and 2E). For example, the F92K mutation (Kabat index) in the light chain (pm3), which is located at the SKY59–C5 interaction site close to T47 and E48 in C5 (Fig 2F), greatly improved the pH dependency. The combination of these mutations did not worsen *k*_a_ at pH 7.4 and provided even better pH dependency (Fig 2D and 2E). The binding kinetics against human C5 was thus drastically improved, and SKY59, which included all these effective mutations, showed high affinity (*K*_D_ = 1.52 × 10^−10^ M) with fast association (*k*_a_ = 1.44 × 10^6^ M^-1^ s^-1^) at pH 7.4 and fast dissociation (*k*_d_ = 1.45 × 10^−2^ s^-1^) at pH 5.8, as previously reported [17]. This C5-binding property of SKY59 allows the engineered antibody to bind strongly to C5 in plasma and release it in the acidic endosome efficiently.

**Table 1:** Mutations in the variable region to improve the C5-binding property.

Mutation	Region	Template	Aim of mutation
am1	CDR-H3	humanized antibody	affinity maturation
am2	CDR-H2	humanized antibody	affinity maturation
am3	CDR-H3	humanized antibody	affinity maturation
am4	CDR-H3	humanized antibody	affinity maturation
am5	CDR-L2	humanized antibody	affinity maturation
am6	CDR-L1	humanized antibody	affinity maturation
pm1	CDR-H2	H/L-AM	pH dependency improvement
pm2	CDR-H3	H/L-AM	pH dependency improvement
pm3	CDR-L3	H/L-AM	pH dependency improvement

Mutations presented in Fig 2 are listed. Mutations am1–am6 were introduced in the humanized antibody to improve C5-binding affinity at neutral pH, while mutations pm1–pm3 were introduced in the affinity-matured antibody H/L-AM to improve pH dependency.

### Effect of the pH-dependent C5-binding property on C5 clearance in mice

**Fig 3:**
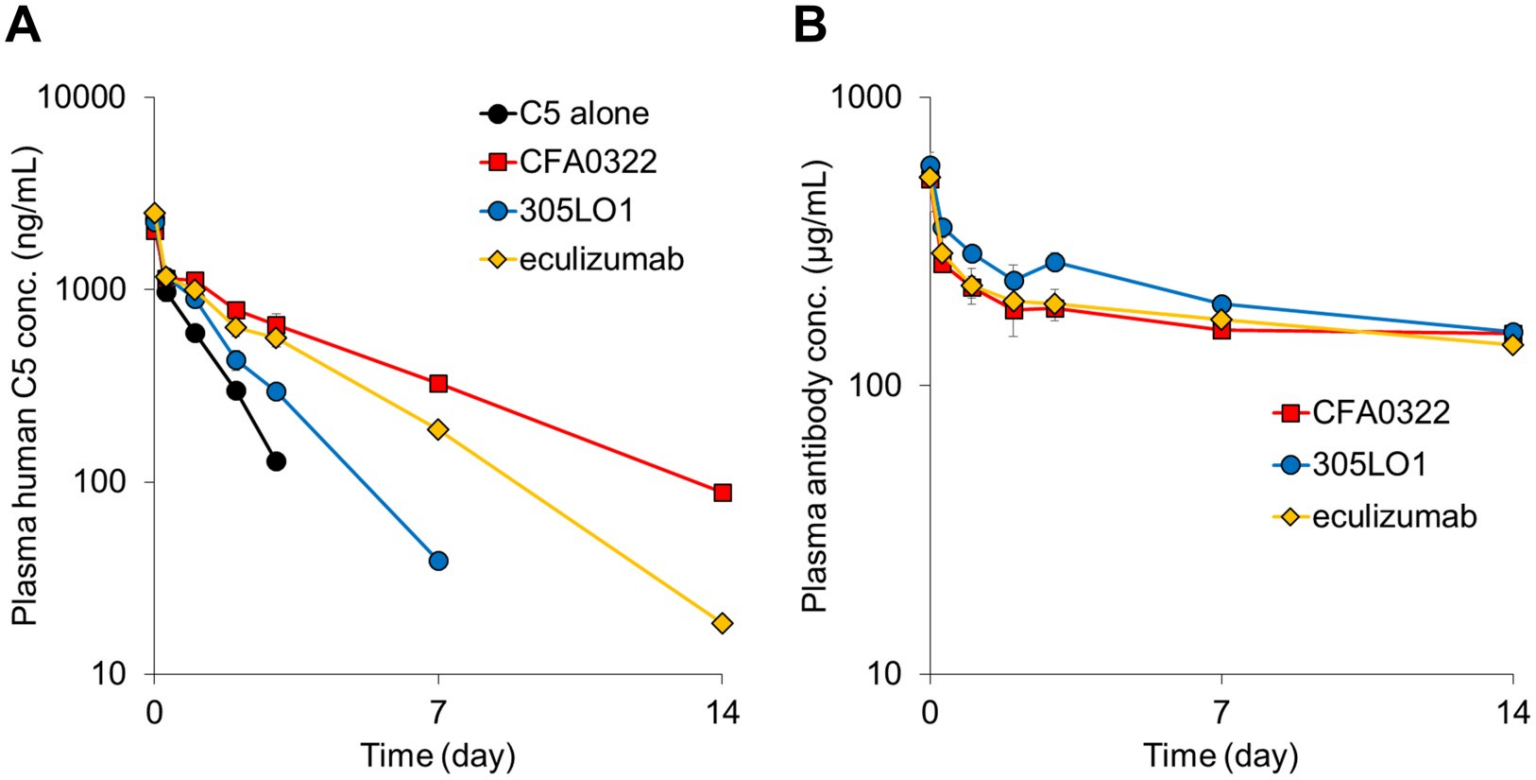
Effect of the pH-dependent C5-binding property on C5 clearance and antibody PK in mice. Human C5 (0.1 mg/kg) with or without an anti-C5 antibody (20 mg/kg) was intravenously administered to human FcRn transgenic mice. Time profiles of human C5 concentration (A) or anti-C5 antibody concentration (B) are shown. Data are presented as mean ± S.D. *n* = 3.

Our previous study has demonstrated that a pH-dependent antigen-binding property increased antigen clearance *in vivo* [11,12]. To confirm the effect of pH dependency on C5 clearance, we conducted a mouse PK study, in which human C5 (0.1 mg/kg) and each anti-C5 antibody (20 mg/kg) were co-injected into human FcRn transgenic mice. CFA0322 is a high affinity antibody without pH dependency that was obtained through rabbit immunization, 305LO1 is a CFA0305 variant with strong pH dependency (Table 2), and eculizumab is an approved high affinity antibody. In this study, CFA0322 and 305LO1 had an engineered human IgG_4_ constant region, while eculizumab had a human IgG_2_/IgG_4_ hybrid constant region [27], neither of which could bind to Fcγ receptors (FcγRs) or C1q. CFA0322 and eculizumab significantly decreased the C5 clearance, but eculizumab had less effect than CFA0322 (Fig 3A). On the other hand, the pH-dependent C5-binding antibody, 305LO1 did not strongly affect the C5 clearance (Fig 3A), and there was no significant difference observed in the plasma concentration of the anti-C5 antibodies at day 14 (Fig 3B). These results demonstrated that pH dependency in the binding to C5 was also effective in suppressing the C5 accumulation that a conventional anti-C5 antibody without pH dependency can cause.

**Table 2:** Variable regions used in the cynomolgus monkey PK study.

Variable region	*K*_D_ at pH 7.4 (M)	*K*_D_ at pH 5.8 (M)	*k*_d_ at pH 5.8 (s^-1^)	Charge engineering
305LO1	5.97 × 10^−9^	3.31 × 10^−5^	4.00 × 10^−2^	not performed
305LO5	4.81 × 10^−10^	1.92 × 10^−7^	1.21 × 10^−2^	not performed
305LO8	1.01 × 10^−9^	5.12 × 10^−7^	3.58 × 10^−2^	performed

Each of three variable regions, 305LO1, 305LO5, 305LO8, was fused with a human IgG_1_ constant region, and the binding kinetics against recombinant cynomolgus C5 were measured by Biacore. 305LO8 was engineered to have an optimal surface charge.

### Effect of the pH-dependent C5-binding property on endogenous C5 accumulation in cynomolgus monkeys

**Fig 4:**
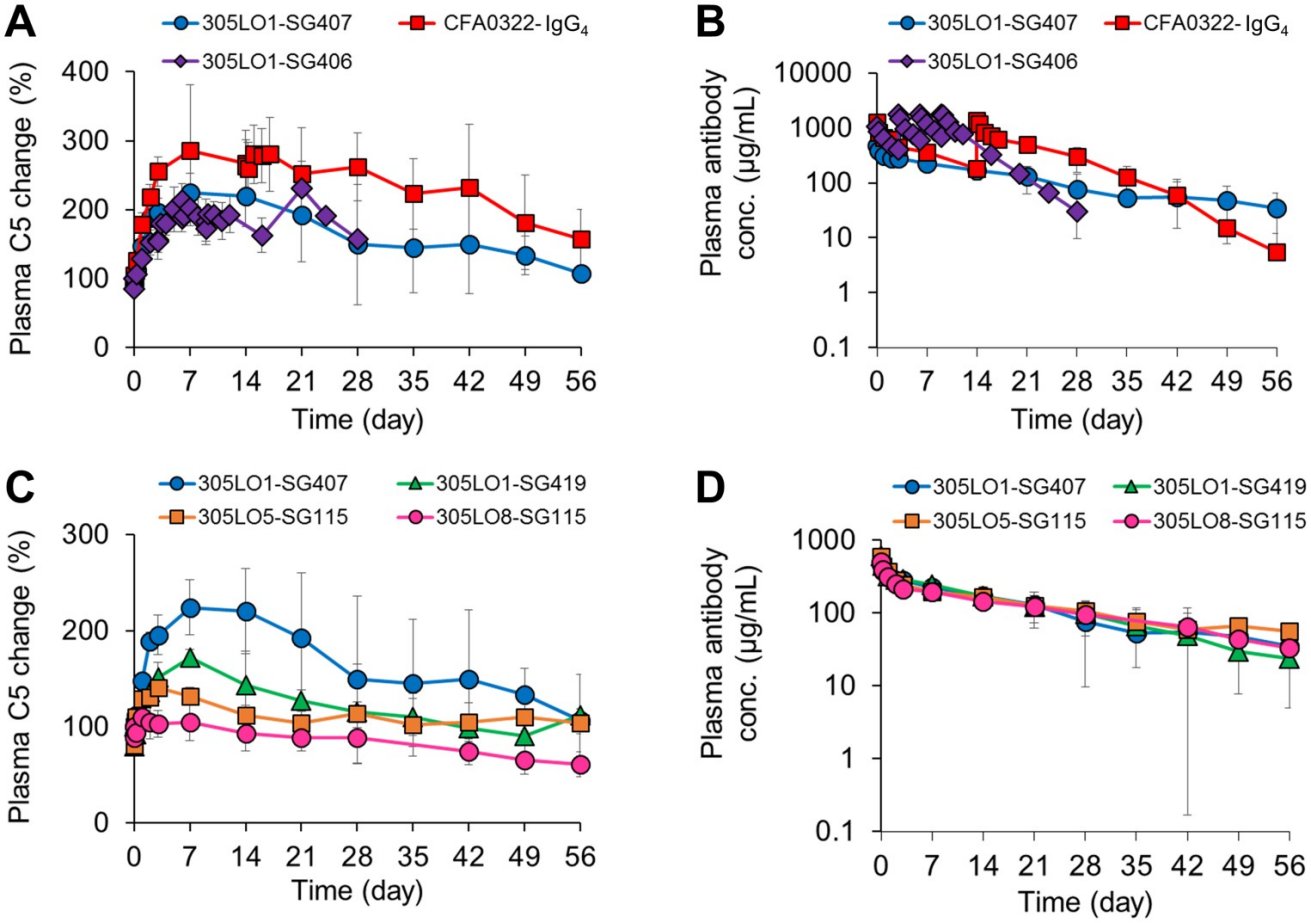
Effect of the pH-dependent C5-binding property and surface-charge engineering on endogenous C5 accumulation and antibody PK in cynomolgus monkeys. Anti-C5 antibodies were intravenously administered to cynomolgus monkeys, and the changes in plasma C5 concentration (A, C) and plasma antibody concentration (B, D) were measured. pH-dependent antibodies with improved FcRn-binding activity, 305LO1-SG407, 305LO1-SG419, 305LO5-SG115, and 305LO8-SG115, were injected at day 0 (20 mg/kg). A pH-dependent C5-binding antibody with no FcRn-binding activity, 305LO1-SG406, was injected at days 0, 3, 6, and 9 (40 mg/kg). A non-pH-dependent C5-binding antibody, CFA0322-IgG_4_, was injected at day 0 and 14 (40 mg/kg). Data are presented as mean ± S.D. *n* = 4. Data points affected by cynomolgus antibodies against the test samples were not included when calculating the mean concentrations.

As 305LO1, with very weak affinity to C5 at acidic pH (Table 2), showed higher C5 clearance in mice compared with the other antibodies (CFA0322 and eculizumab), we evaluated the effect of pH dependency on endogenous C5 accumulation in cynomolgus monkeys using antibodies with various properties in their variable and constant regions (see Tables 2 and 3, respectively). CFA0322 was used as the non–pH-dependent C5-binding antibody because eculizumab has minimal activity against any other primate or mammalian C5 [27], and we found that the non–pH-dependent antibody increased the concentration of endogenous C5 in plasma to 286% compared with the values at pre-administration (Fig 4A). As for pH-dependent antibodies, we first administered 305LO1-SG407, which has the 305LO1 variable region with weakened affinity at acidic pH and a constant region with enhanced FcRn-binding activity at acidic pH [32], and found that the antibody increased plasma endogenous C5 concentration to 224% (Fig 4A). We next examined 305LO1-SG406, which consists of the same 305LO1 variable region and an engineered constant region with no FcRn-binding activity. Since this non–FcRn-binding antibody also accumulated plasma endogenous C5 to the same level as the FcRn-binding antibody (Fig 4A), we assumed that a slower nonspecific uptake rate of the C5-antibody complex than C5 alone would be the main mechanism of C5 accumulation by the pH-dependent antibodies.

**Table 3:** Constant regions used in the cynomolgus monkey PK study.

Constant region	Backbone	FcRn binding	FcγR & C1q binding	pI
SG406	human IgG_4_	silenced	silenced	low
SG407	human IgG_4_	enhanced	silenced	low
SG419	human IgG_4_	enhanced	silenced	high
SG115	human IgG_1_	enhanced	silenced	high

The constant region of human IgG_4_ or IgG_1_ was engineered in this study. In SG406, FcRn binding was abrogated to prevent the antibody being salvaged back to plasma from the endosomes by FcRn-mediated recycling. In SG419, human IgG_4_ was engineered to increase the pI. The surface-charge engineering was not incorporated in SG115, because the human IgG_1_ backbone naturally had high pI.

### Surface-charge engineering in the variable and constant regions to further suppress C5 accumulation

Because nonspecific clearance from blood circulation is known to be correlated with the pI of proteins [38], we tested 305LO1-SG419, which has the 305LO1 variable region and a constant region that has been engineered to give a surface charge with higher pI (Tables 3 and 4). The C5 accumulation was significantly suppressed when the SG419 constant region with higher pI was used, while the surface-charge engineering did not clearly affect the concentration of the antibody in plasma (Fig 4C and 4D). Furthermore, we examined two other engineered derivatives, 305LO5-SG115 and 305LO8-SG115, both of which have pH dependency but slightly different pI in their variable region and an identical constant region with high pI (Tables 2–4). In line with the observation above, the higher-pI variant 305LO8 exhibited less accumulation of endogenous C5 in plasma with no significant difference in the antibody clearance (Fig 4C and 4D), suggesting that surface-charge engineering to increase the pI of both constant and variable regions of the antibody could suppress antigen accumulation.

**Table 4:** Charge-related properties and C5 accumulation in cynomolgus monkeys.

Antibody	pI of antibody	CIEX RT of IC-1 (min)	Maximum C5 accumulation (%)
305LO1-SG407	8.18	22.8	224
305LO1-SG419	9.23	27.5	172
305LO5-SG115	9.23	32.7	141
305LO8-SG115	9.45	33.7	111

**Fig 5:**
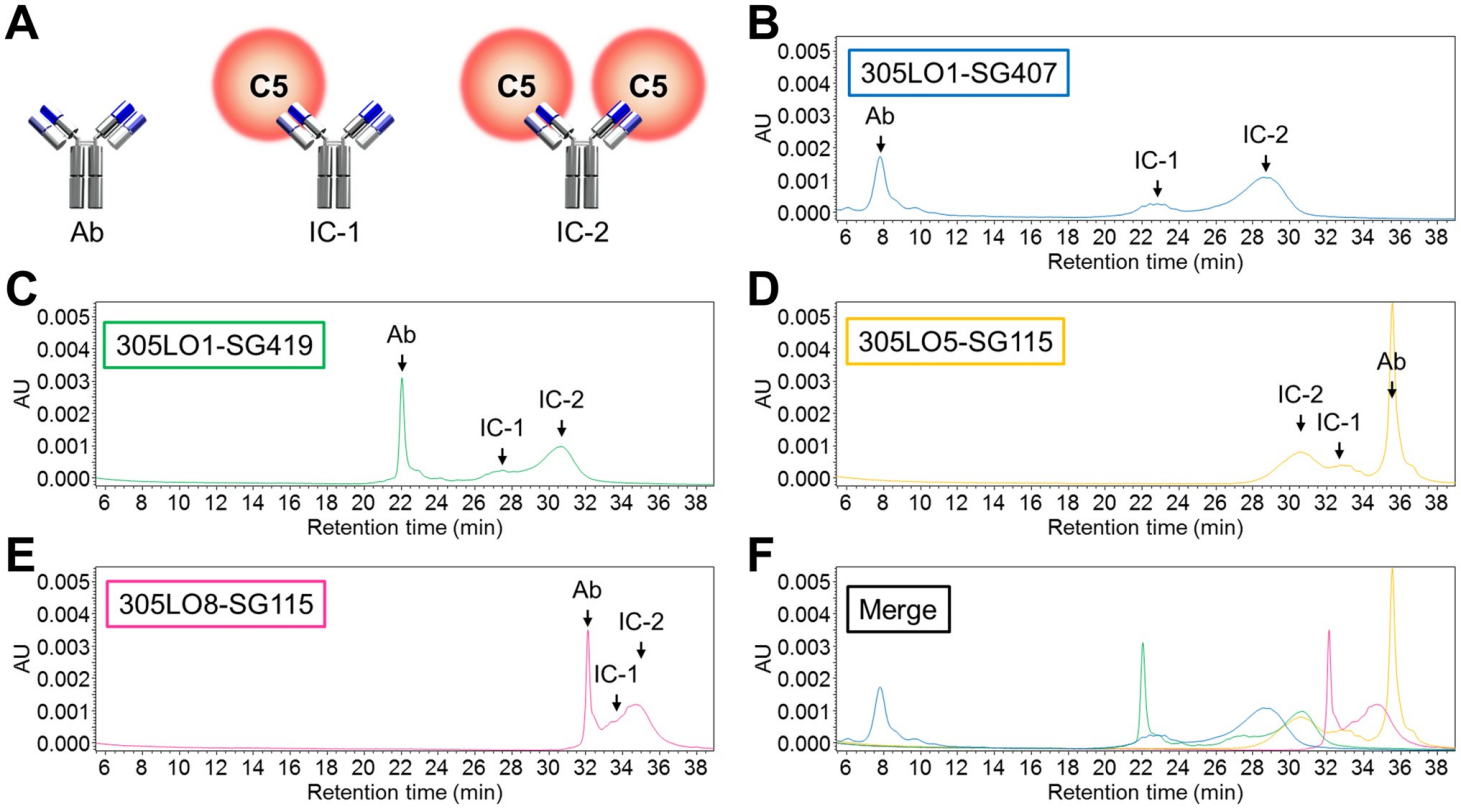
CIEX analysis of C5–antibody complexes. When an antibody that recognizes a specific epitope in C5 is administered, two types of IC can form (A). A CIEX chromatography analysis was performed for the mixture of cynomolgus C5 and each anti-C5 antibody: 305LO1-SG407, 305LO1-SG419, 305LO5-SG115, and 305LO8-SG115 (B‒F).

The table shows the experimental pI of each antibody and the retention time in a CIEX analysis of IC-1, which consists of one C5 and one antibody (as depicted in Fig 5A). The mean value of maximum C5 accumulation was obtained from the cynomolgus monkey PK study.

### CIEX analysis to predict antigen accumulation *in vivo*

**Fig 6:**
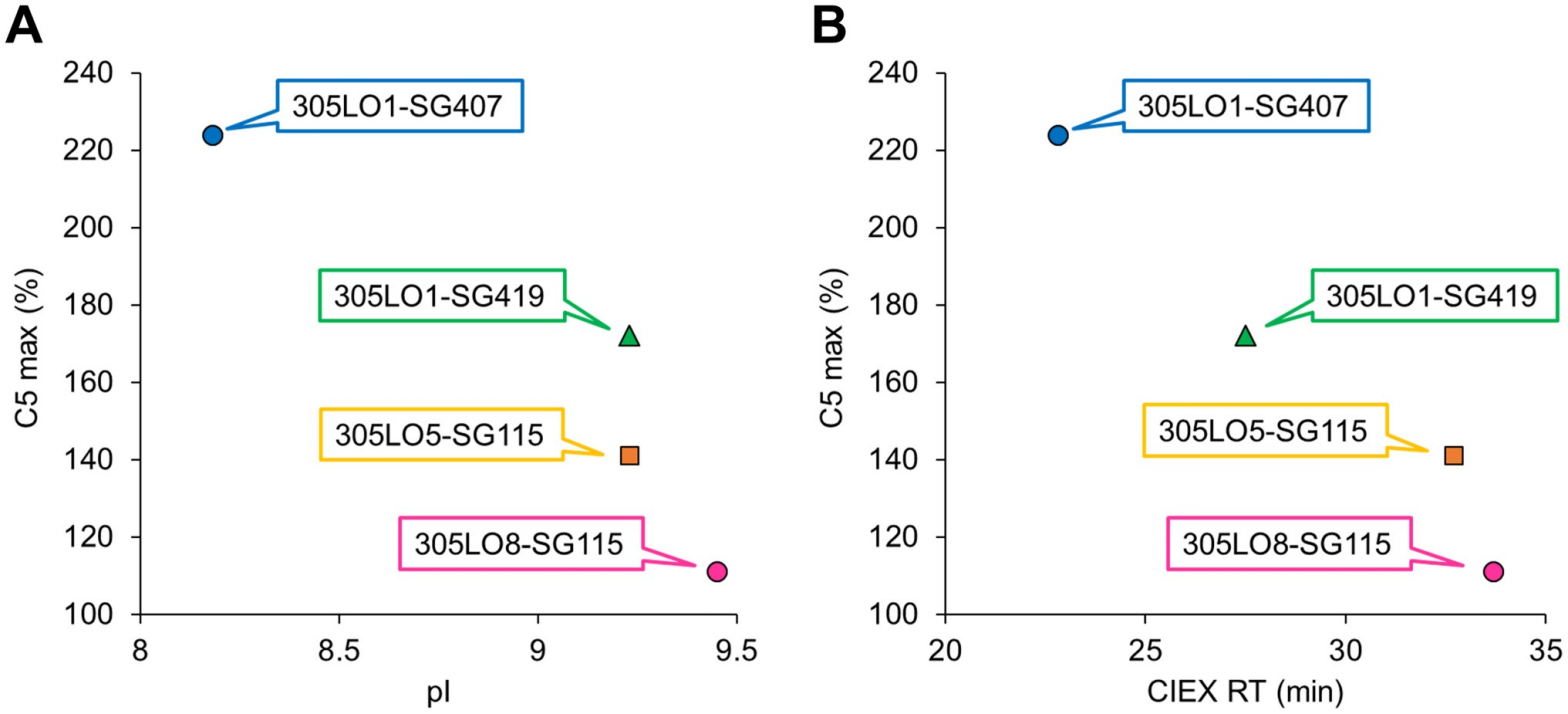
Correlation between C5 accumulation and pI of the antibody or CIEX retention time of the IC. The correlation between C5 accumulation and the experimental pI of each antibody (A) or the retention time of IC-1 (which consists of one C5 and one antibody) in CIEX analysis (B) was analyzed.

Whilst the result of the PK study suggested that an antibody variant with higher pI accumulated C5 less, we actually considered the pI or surface charge of an IC during the optimization because it is more likely to affect the C5 clearance. Thus, in addition to measuring the pI of each antibody, we conducted a CIEX chromatography analysis of the C5–antibody complexes to understand the relationship between charge and C5 accumulation (Table 4 and Fig 5). When an excess amount of the anti-C5 antibody was administered in the PK study, most C5 proteins formed an IC consisting of one C5 and one antibody; therefore, we measured the retention time of that type of IC (IC-1). The maximum accumulation of C5 in the cynomolgus monkey PK study seemed to correlate with the pI of the antibodies and the retention time (RT) of IC-1 in the CIEX analysis; however, it was only from data in the CIEX analysis that the difference between 305LO1-SG419 and 305LO5-SG115 could be predicted (Fig 6).

### Other antibody engineering to improve SKY59’s properties

**Fig 7:**
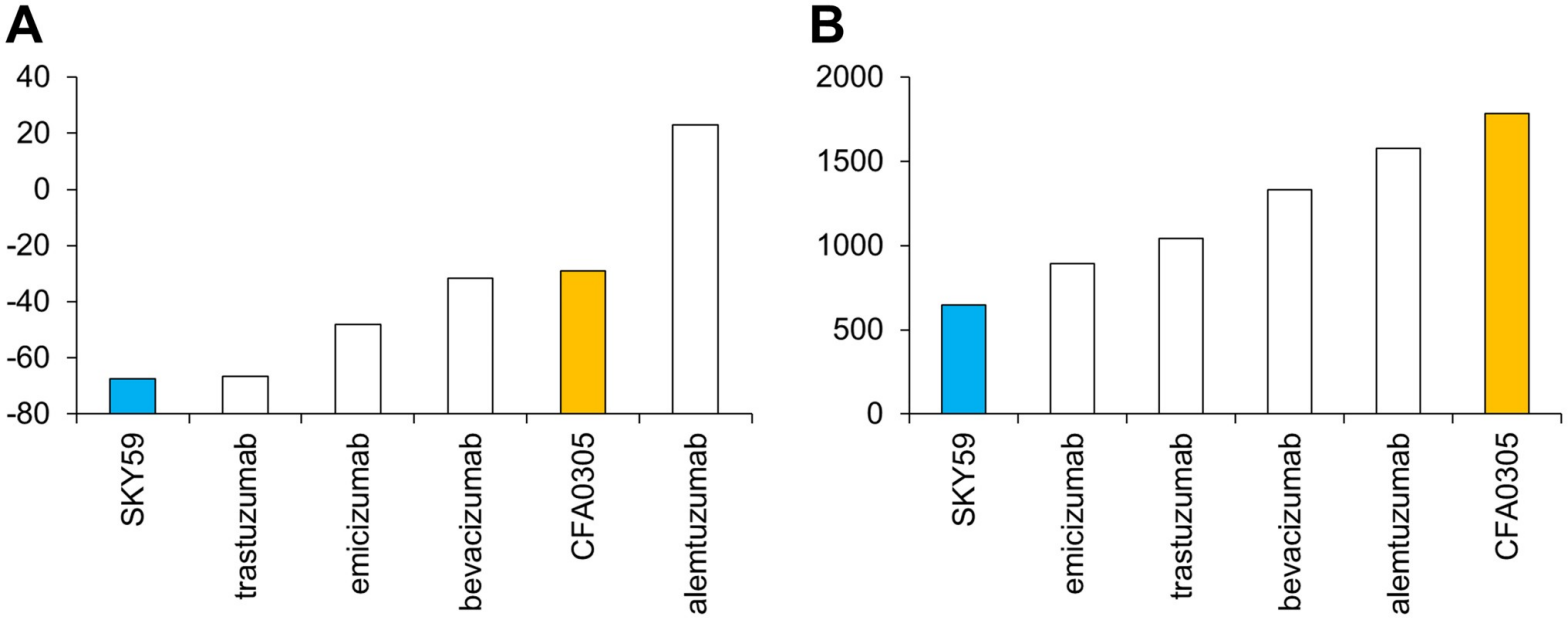
Potential immunogenicity of antibodies predicted *in silico*. Potential immunogenicity of SKY59 (light blue), the lead antibody CFA0305 (orange), and other approved antibodies was evaluated *in silico*. Antibodies with a lower Tregitope-adjusted EpiMatrix score (A) or a lower Epibase immunogenicity risk score (B) are considered to be less immunogenic.

Fig 1 lists the further optimizations that were conducted for the clinical application of SKY59. In order to minimize the number of potential immunogenic T cell epitopes, we substituted CDR residues referring to human germline sequences to make the CDRs or CDR/framework junction regions more human-like. In addition, we introduced mutations that can reduce immunogenicity scores, as calculated by two types of *in silico* analysis [33–36]. The humanized and further deimmunized antibody SKY59 was found to have a comparable or smaller risk of immunogenicity compared with other approved humanized antibodies (Fig 7). The human IgG_1_ κ constant region was engineered to silence the binding to FcγRs and C1q, to minimize the potential safety risk of activating effector functions. The binding to FcRn was moderately enhanced to improve the PK of the antibody, whilst binding to rheumatoid factor (RF) was suppressed [32]. To reduce C-terminal heterogeneity of the antibody heavy chain [39,40], the two C-terminal amino acids, glycine and lysine, were genetically removed. Viscosity and overall stability of the antibody for manufacturing and liquid formulation were also monitored periodically during the optimization described above. The engineered clinical candidate antibody, SKY59, has good manufacturability and can be developed as a liquid formulation with high antibody concentration for subcutaneous delivery.

## Discussion

### Humanization and cysteine residues in CDRs

When anti-drug antibodies are produced in patients, the clinical use of therapeutic antibodies becomes limited; therefore, humanization to minimize the potential risk of immunogenicity is an essential antibody engineering technology. In this report, we humanized the rabbit antibody CFA0305 using framework shuffling [37]. Although cysteine residues are rare in the CDRs of human antibodies, some rabbit antibodies contain cysteine residues in their CDRs, and we also found them in the CDRs of CFA0305. The cysteine residue found in CDR-L3 was considered to be unpaired, which would cause dimers, oligomers, aggregations, or other sources of heterogeneity in the manufacturing process, resulting in a higher risk of potential issues at the Chemistry, Manufacturing, and Controls (CMC) stage or in immunogenicity [39,41,42]. The cysteine residues in CDR-H1 and CDR-H2, on the other hand, were able to form an intra-domain disulfide bond. However, incomplete formation of the disulfide bond may also cause CMC issues in large-scale manufacturing [39,41], and could provide immunogenic T cell epitopes, as they are not found in human germline sequences. Thus, we substituted them with alanine or serine in the humanization process. Although the engineering that reduced immunogenicity or removed cysteine residues affected the C5 binding activity, the drop in binding activity was recovered by applying effective mutations identified in the subsequent comprehensive mutagenesis.

### Mutations that improved the C5-binding property

A number of antibodies have been successfully engineered to have a preferable pH-dependent antigen-binding property by the histidine scanning approach, in which we systematically substitute key residues with histidine and combine effective mutations [11,18–20]. Alternatively, combinatorial histidine scanning libraries have conferred pH-dependent antigen binding on lead antibodies [21,22]. In addition, pH-dependent antibodies have been identified directly from a histidine-rich library [23], and also generated naturally in animals [17,24,25]. As we have reported, the pH-dependent interaction between C5 and SKY59 was partly due to histidine residues found in the epitope on C5 [17]. This would explain the fact that the lead antibody for SKY59 was obtained from immunized rabbits and had a pH-dependent antigen-binding property without engineering, even though only one histidine residue was found in its variable region. Our screening result suggests that H100c, the histidine residue initially existing in the CDR-H3, contributes to the pH dependency presumably because when the histidine residue is protonated under acidic conditions, it can induce an electrostatic repulsion between the lysine at position 109 and the histidine at position 110 in C5 (Fig 2F). We found a few non-histidine mutations that improved the pH-dependent C5-binding property (Fig 2E), though no such mutation has yet been reported. In the light chain, the F92K mutation greatly improved the pH dependency, supposedly because the F92K mutation interfered somewhat with the hydrogen-bond interaction between H100c of the heavy chain and the glutamic acid at position 48 in C5, which weakened the interaction and improved the effect of H100c on pH dependency (Fig 2F). This study demonstrates that we could identify non-histidine mutations that enhance the pH-dependent interaction initially given by histidines in the antibody or its antigen. These non-histidine mutations would be helpful to achieve a challenging binding property, such as that of extremely high affinity at neutral pH and fast dissociation at acidic pH, which is sometimes required to display a clear advantage for patients in clinic over conventional high-affinity antibodies. Comprehensive mutagenesis would be an efficient way to identify such mutations and further improve the pH dependency.

### pH-dependent C5-binding property increases C5 clearance and also improves antibody PK *in vivo*

Our mouse PK study showed that 305LO1 with sufficiently weakened C5-binding activity at pH 5.8 did not reduce C5 clearance as strongly as the other antibodies did (Fig 3A). Interestingly, C5 clearance was lower with non–pH-dependent CFA0322 than with eculizumab, which can be explained by the difference in the clearance of their ICs. In a separate mouse PK study, a high concentration of exogenous human C5 accelerated the clearance of eculizumab, possibly because the eculizumab–C5 IC has a lower capacity to bind to FcRn in endosomes or is actively cleared from the blood circulation by another mechanism not yet elucidated [20]. Actually, binding to a soluble antigen sometimes lowers the FcRn-binding capacity of ICs or accelerates antibody clearance that is target-dependent. For example, antibodies targeting human TNFα or IL-10 were reported to have reduced binding to FcRn when conjugated to the antigen [43,44], and anti-PCSK9 antibodies showed increased *in vivo* clearance dependent on PCSK9 [18]. We assume that a lower FcRn-binding capacity or an unknown mechanism of accelerated uptake of the eculizumab–C5 IC made C5 clearance higher than with CFA0322. As for antibody clearance, there was no clear difference among the antibodies tested in our mouse PK study because antibody concentration was higher than C5 in plasma (Fig 3B). When the antibody concentration is much higher than C5 concentration, target-mediated degradation increases the clearance of C5. Whilst target-mediated degradation of the IC can suppress antigen accumulation, inefficient FcRn-mediated salvage or accelerated uptake of the antibody normally requires a higher dosage for treatment. In contrast, a pH-dependent C5-binding property was expected to increase C5 clearance and maximize the capacity of C5-free antibodies to bind to FcRn for efficient recycling. One of the engineered derivatives of SKY59 evaluated in this project, termed CFA0305 (NpH)-IgG_4_, had strong affinity to C5 under both neutral and acidic pH conditions [17]. In the cynomolgus monkey PK study, the clearance of this non–pH-dependent antibody was accelerated when the antibody concentration became low, which we assume was because the IC had a lower FcRn-binding capacity than the antigen-free antibody. This means that even if an antibody with insufficient pH dependency forms ICs that have a low FcRn-binding capacity, the salvage efficiency of the antigen-free antibody by FcRn can be recovered by conferring sufficient pH dependency on the antibody, as demonstrated by SKY59.

### Endogenous C5 accumulation by pH-dependent antigen-binding antibodies in monkeys

It was recently reported that an engineered eculizumab variant, ALXN1210, was obtained by introducing histidine residues for pH-dependent C5 binding in the variable region of eculizumab and by engineering its Fc portion to enhance FcRn binding [20]. Because eculizumab has minimal activity against any other primate or mammalian C5 [27], no monkey PK study was described. Instead, the authors demonstrated the improved plasma half-life of ALXN1210 relative to eculizumab in NOD-SCID mice administered with a high concentration of exogenous human C5. However, the effect of the pH-dependent C5-binding antibody on C5 clearance was not described, which must be considered when even longer-lasting neutralization is desired. In our case, we evaluated the PK of our antibodies and the accumulation of endogenous C5 in plasma in cynomolgus monkeys, since the PK of therapeutic antibodies in human can be quantitatively predicted from that in cynomolgus monkeys [45]. The PK study clearly demonstrated that it was difficult to suppress antigen accumulation completely by just engineering to lower the antigen-binding affinity at acidic pH. Despite the very weak C5-binding activity at acidic pH of 305LO1 (Table 2), 305LO1-SG407 accumulated endogenous C5 in cynomolgus monkeys (Fig 4A). To clarify the mechanism by which a pH-dependent C5-binding antibody accumulates C5, we examined 305LO1-SG406, which has no FcRn-binding activity (Table 3). When non–FcRn-binding antibodies are administered, neither antigen nor antibody taken up by cells can be recycled back to plasma by FcRn. Therefore, the antigen clearance by non–FcRn-binding antibodies should be comparable with that by antibodies with sufficient pH dependency to completely release the antigens in endosomes. If the pH-dependent dissociation of 305LO1-SG407 from C5 was insufficient, it would have accumulated more C5 than 305LO1-SG406. Since the two antibodies showed similar levels of C5 accumulation (Fig 4A), 305LO1 was considered to have sufficient pH dependency and to completely release C5 in endosomes. The result also means that even when an antibody completely dissociates C5 in acidic endosomes and there is no salvage of C5 by FcRn, plasma C5 concentration still increases.

### Antibody engineering to suppress endogenous C5 accumulation

The C5 accumulation by the pH-dependent antibodies can be explained by a lower nonspecific uptake rate of C5 from blood circulation into cells when C5 is captured by the antibody. We hypothesized that increasing the pI or optimizing the surface charge of the IC could enhance the uptake because antibodies with higher pI were reported to display larger clearance [38,46]. Although we were concerned that this modification might shorten the plasma half-life of the antibody, we presumed that the engineering to enhance FcRn binding at acidic pH would maximize the salvage efficiency from the endosome and minimize the negative effect on the clearance of the antibody. Initially, human IgG_4_ was used because of its low potential of effector functions, but we engineered the human IgG_4_ constant region to have higher pI by using segments of human IgG_1_ to minimize the risk of immunogenicity. As we expected, the PK study suggested that the IgG_4_ constant region variant with higher pI showed less C5 accumulation in cynomolgus monkeys (Fig 4C). Since human IgG_1_ naturally has higher pI than IgG_4_, we then also examined a variant of human IgG_1_, which was engineered to bind to neither FcγRs nor C1q. Using the human IgG_1_ constant region variant, surface-charge engineering on the variable region seemed also effective to suppress C5 accumulation (Fig 4C), although 305LO8 showed weaker affinity at pH 5.8 than 305LO5 (Table 2). Especially when engineering the variable region, we carefully considered positions for substitutions. If a mutation that increases pI, for example a positively-charged residue, is introduced in the antigen-contacting region, the mutation on the C5-binding arm of the antibody would be ineffective because it is buried by C5, possibly resulting in just a shorter plasma half-life of the antibody and inefficient uptake of the IC. On the other hand, pI-decreasing mutations in the antigen-contacting region could provide a longer plasma half-life of the antigen-free antibody with a minimum effect on IC uptake. Thus, we analyzed antibody variants using CIEX analysis in the presence of C5 and monitored the retention time of ICs (Fig 5), so that we could predict the effect of each mutation on the C5-free antibodies as well as on ICs (Fig 6). Samples in the CIEX analysis were prepared so that sufficient amounts of IC-1 and IC-2 complexes and C5-free antibodies could be detected (see Materials and methods). Because an excess amount of the injected antibody, as when administered in the PK study, results in most C5 proteins forming IC-1 in plasma, we monitored IC-1 retention time to investigate C5 accumulation. For engineering purposes, however, IC-2 is also relevant because, as antibody concentration decreases, the IC-2 complex occurs more frequently and thus affects changes in C5 concentration in plasma. Fortunately, the surface-charge engineering did not strongly affect the plasma half-life of the antibody (Fig 4D), conceivably because the mutation to enhance the FcRn binding at acidic pH maximized the efficiency of salvaging the antibodies in endosomes by FcRn.

There are other types of engineering that can be used to further enhance the antigen clearance, some of which could also be applied for anti-C5 therapy. Although we moderately enhanced the FcRn binding at acidic pH to lengthen the plasma half-life of SKY59, Fc modification to further enhance FcRn binding generated an antibody that binds to FcRn at neutral pH, which increased the antigen clearance more efficiently [12]. FcγRIIb-mediated sweeping can also be applied to increase antigen clearance [14]. Although C5 uptake can be enhanced just by increasing the binding activity of the antibody to Fc receptors, this could also increase the clearance of antigen-free antibodies. Instead, a biparatopic antibody that recognizes two different epitopes on the antigen forms larger ICs containing multiple antibodies that can be efficiently taken up by FcγR-expressing cells by the avidity effect [47]. Therefore, a pH-dependent biparatopic antibody can provide a much more efficient way of sweeping C5 from the blood, because only ICs are quickly taken up, while antigen-free antibodies are not affected [48]. However, manufacturing a biparatopic antibody is more complicated and needs further engineering and, in addition, the possible effects of FcγR binding or large IC formation in patients must be carefully considered before its clinical application. Surface-charge engineering is a novel antibody technology that can be widely used and combined with other engineering technologies to maximize the neutralization capacity of recycling and sweeping antibodies.

### Improved properties of SKY59

During this optimization process, we improved various properties of the antibody by substituting amino acid residues. A common setback in multidimensional optimization is that a substitution that effectively improves one property often worsens other properties. For example, a mutation that improves antibody PK or suppresses C5 accumulation can weaken the C5-binding property. Our comprehensive mutagenesis method, termed COSMO, provides information about the effects of all possible mutations on antigen-binding activity, by which we can more smoothly optimize our antibodies. This enabled us to successfully generate SKY59, which exhibits an improved C5-binding property, a long plasma half-life with lower C5 accumulation, high stability, and less immunogenicity predicted *in silico*. Immunogenicity is a concern with a highly engineered antibody. Fortunately, two different *in silico* systems predicted SKY59 to have low immunogenicity. Although the true immunogenicity of SKY59 has yet to be evaluated clinically, it is noteworthy that emicizumab, also known as ACE910, which was deimmunized in the same way [49], showed low immunogenicity in patients [50,51]. The engineered antibody, SKY59, presents a long-acting neutralizing capacity in cynomolgus monkeys [17], and its high stability allows it to be formulated at a high antibody concentration in a liquid formulation for subcutaneous delivery.

## Conclusions

We have generated a novel anti-C5 recycling antibody through rabbit immunization and multidimensional optimization. The property to have high affinity binding to C5 at neutral pH and fast dissociation at acidic pH was achieved by targeting an epitope on C5 that was good for pH dependency and engineering through the COSMO approach. Our monkey PK study demonstrated that although pH-dependent antibodies accumulated C5, this action was suppressed by engineering the surface charge of the antibody. The antibody engineering performed in this study can be used on various recycling and sweeping antibodies and provides a superior capacity for antigen neutralization, leading to even smaller doses that allow subcutaneous delivery and/or less frequent injections. SKY59 is an engineered IgG_1_ antibody with a humanized variable region belonging to the human V_H_3-V_H_2/V_κ_1 subfamily and is expected to improve the quality-of-life of patients with complement-mediated disorders. SKY59 is currently being evaluated in clinical studies [52].
